# Supplementary material for: Proteomic Characterization of PAMs with PRRSV-ADE Infection
Source: Viruses. 2022 Dec 22;15(1):36. doi: 10.3390/v15010036 (PMC9864506; doi:10.3390/v15010036)
Supplement: Supplementary file 1 [file viruses-15-00036-s001.zip › viruses-2106570-supplementary.pdf]

Table S1. The primers used in this study

Primer Name	Primer Sequence (5' -3')
Actin	Forward: CGGGACATCAAGGAGAAGC
	Reverse: CTCGTTGCCGATGGTGATG
COX-5B	Forward: GGGACTGGACCCATACAATATAC
	Reverse: CACAGATGCAGCCCACTAT
ISG56[9]	Forward: TCAGAGGTGAGAAGGCTGGT
	Reverse: GCTTCCTGCAAGTGTCCTTC
MMP-9	Forward: CGACATCTTCCAGTACCAAGAG
	Reverse: CCCACATAGTCCACCTGATTC
Mx1	Forward: TACGACATCGAATACCAGATCAA
	Reverse: ATGGTCCTGTCTCCTTCGG
OAS1	Forward: CATCAGAAGCTTTGCATCT
	Reverse: GGCCTGGGTTTCTTGAGTT
RSAD2	Forward: GCGCTTCCTGAACTGTAGAA
	Reverse: TCCTCCTCGCTTTAGAAACATC
